# Hospitals under 150 Beds

**Published:** 1918-12-21

**Authors:** 


**Hospital Needs and Hospital Appeals**—(*continued*).

### Hospitals under 150 Beds.

**THE CANCER HOSPITAL, FULHAM ROAD, S.W. 3.**—On the commencement of the war the committee offered the War Office, and the offer was accepted, the absolutely free use of fifty beds and of the out-patient and electrical and radio-therapeutic departments, and much good work has been carried out, especially in the latter department, where many hundreds of service men have been diagnosed or treated, and many cases have thus been returned to military duties, or useful civil occupations. At present the whole of the 127 beds available are occupied by civilians. The staff of the hospital are considering several questions for the improvement of the facilities of the hospital, the chief being the provision of second operating theatre, and the extension of the work of the electrical and therapeutic department, but all these questions must depend on the support given, as at present the ordinary income of the hospital falls short by some £8,000 to £10,000 a year of the expenditure, and the committee are forced to draw on the capital account or make use of the legacies to provide the deficit.

**CHELSEA HOSPITAL FOR WOMEN.**—Early in the New Year the wards of this hospital as far as circumstances permit will again be devoted exclusively to the treatment of the diseases to which women particularly are liable. Over 400 wounded officers have been received during the past fifteen months, and a very appreciative letter has been received from the D.D.M.S. in reference to the value of the services so rendered to them. These demands being satisfied twenty-five additional women patients can be admitted. When funds are available for building a new nurses' home, and for maintaining the patients who will then be able to occupy the beds now used for nurses, the full value of the hospital to suffering women will be realised. Contributions will be gratefully received by the Hon. Treasurer, Mr. Sidney H. Goldsmid, or the Secretary, Mr. H. H. Jennings, at the Hospital, Arthur Street, Chelsea, S.W. 3.

**EAST LONDON HOSPITAL FOR CHILDREN, SHADWELL, E. 1.**—1918 is the Jubilee year of the hospital, and a fund to be known as the Jubilee Fund has been opened, the organisation of which is being carried out in a West End office at 199 Piccadilly. The organising secretary is Mrs. Sargon. The needs of the hospital are just as great as ever, and in spite of much liberality from many subscribers and donors it is still a struggle to make both ends meet and to keep clear of debt. There is no use dwelling on the importance of this work as it is patent to everyone. The Jubilee Fund will remain open all next year, and it is hoped that still more liberal support from all classes to enable us to carry on this very essential work will be forthcoming. The secretary is Mr. W. M. Wilcox.

**HOSPITAL AND HOME FOR SICK CHILDREN, SYDENHAM.**—The work of this children's hospital, which has sixty cots, has been maintained all through the four years of war as in peace time, but unfortunately the immense increase in the cost of necessary supplies has forced the committee to borrow £1,000. Funds are urgently needed to liquidate this debt and for future maintenance. A new laundry and a system of central heating are badly needed. Hon. Treasurer, Mr. P. W. Killby.

**HOSPITAL FOR EPILEPSY AND PARALYSIS.**—During the course of the last half-century the staff of the Hospital for Nervous Diseases, Maida Vale, have been acquiring that knowledge and skill which is enabling them to do so much for war wrecks both at Maida Vale and at the Golders Green branch. There are eighty-five beds at Maida Vale, including thirty-five for war wrecks from the trenches, and twenty-five for sailors, and 120 beds at Golders Green for Royal Air Force flying officers suffering from nervous strain. The hospital has an overdraft at the bank of £3,500. Mr. H. W. Burleigh is the Secretary.

**THE INFANTS' HOSPITAL, VINCENT SQUARE, WESTMINSTER, S.W. 1.**—The work of this hospital in saving infant life is now of greater importance than ever; but the hospital is entirely without funds for maintenance, and in debt to the extent of £4,000—a much larger sum than at any previous time. The Secretary is Mr. Alfred J. Small.

**MILDMAY MISSION HOSPITAL, AUSTIN STREET, BETHNAL GREEN, E. 2.**—For upwards of forty years this hospital has carried on a much needed work amongst the sick poor of the East End of London, a large proportion of the patients being of the Jewish class. The struggle during the past four years to carry on has been severe. Christmas contributions should be addressed to the Secretary, Miss M. A. Bellamy.

**Hospital Needs and Hospital Appeals—(*continued*).**

**MOUNT VERNON HOSPITAL FOR CONSUMPTION, NORTHWOOD, and FITZROY SQUARE, LONDON, W.,** is in great need of annual subscriptions and donations. Owing to the increased cost of maintenance, the expenditure is greatly in excess of the income for the current year. The treatment of women and children is a special feature. A large number of discharged soldiers and prisoners of war are received, and many cases have been referred by the military authorities for special attention in the out-patient department during this year. Mr. W. J. Morton, Secretary.

**NATIONAL HOSPITAL FOR DISEASES OF THE HEART, WESTMORELAND STREET, LONDON, W. 1.**—The work has expanded greatly since the hospital was rebuilt, and it is hoped that some extension may be possible in the future. It may fairly be claimed that the hospital has rendered useful service during the war by the examination of upwards of 12,000 recruits in regard to which the Ministry of National Service has expressed appreciation, and by the treatment of discharged disabled sailors and soldiers. The Chairman is Sir James Harrison, C.V.O., who is carrying out the duties of Secretary during the absence of Mr. Robert Whitney on active service.

**POPLAR HOSPITAL FOR ACCIDENTS.**—The hospital issues a leaflet containing reasons for helping, amongst which is the fact that the institution is situated in the centre of the Port of London, and is in the best possible position for dealing with the serious accidents—owing to the war pressure, more numerous and serious than ever—which occur at the docks and at the hundreds of factories in the East End of London. Funds for maintenance are asked for. Mr. Percy Rogers is the Secretary.

**QUEEN CHARLOTTE'S LYING-IN HOSPITAL, MARYLEBONE ROAD, N.W. 1.**—On the outbreak of war an extension of the hospital (for new out-patient department, new kitchens, lecture-room, pathological laboratory, etc.) was about to be undertaken, but had to be abandoned for the time. King Edward's Hospital Fund has approved the scheme, and has made grants towards the cost, and it is hoped to be able to proceed with the extension at an early date. Meanwhile temporary premises for the important ante-natal and infant welfare work have been provided adjoining the hospital. Mr. Arthur Watts is the Secretary.

**QUEEN'S HOSPITAL FOR CHILDREN, HACKNEY ROAD, BETHNAL GREEN, E. 2.**—Since the outbreak of the war in August 1914 the work of this hospital for children has been carried on under the most trying conditions, but thanks to the generosity of the British public and to the devotion of the medical and nursing staffs, more than 8,000 children have been treated in its wards, while nearly half-a-million attendances have been made by poor little suffering out-patients. It need hardly be pointed out that as a result numbers equalling the population of a large city have been restored to health to become in due course a potential asset to our country. The committee are now faced with an immediate deficit of £6,000 in respect of maintenance. This sum must be raised by the end of the year if the hospital is to start 1919 with a fair chance of continuing to deal with all the claimants for relief. Mr. T. Glenton-Kerr is the Secretary.

**THE ROYAL WATERLOO HOSPITAL, WATERLOO ROAD, S.E. 1.**—It is proposed to rebuild the old and unsuitable nurses' quarters, and general extension and improvement as soon as conditions permit. The assured income is only about £3,000 to meet an annual expenditure of £11,000. The hospital could not undertake military cases, as the work among the sick children of the poor was considered of first-rate national importance. The hospital has been a factory for munitions of life. Mr. Alexander Pym, Manager.

**ST. MARK'S HOSPITAL, CITY ROAD.**—The greatest need at present is the help of the charitable public in securing the necessary funds to continue the work in curing and relieving a large number of cancer and other rectal cases. During the year the committee have acquired the vacant freehold site adjoining the hospital. A very grave danger was threatened by the proposed erection of a factory. Increased generous help is asked for. The Secretary is Mr. H. W. G. Coope.

**SOUTH LONDON HOSPITAL FOR WOMEN.**—This hospital has eighty beds for women and children, and is entirely officered by medical women. A special feature is the provision of private wards for women of limited means at low inclusive fees. By arrangement with the War Office one of these wards has, for some time past, been set apart for the use of general service V.A.D. patients requiring treatment in hospital. The work at the out-patient department, situated at 86-90 Newington Causeway, continues to increase, and owing to the restricted accommodation numbers of patients have to be turned away daily. The board of management earnestly appeal for contributions and special Christmas gifts, which will be gladly received by the Secretary at the Hospital, South Side, Clapham Common, S.W. 4.

**WEST END HOSPITAL FOR NERVOUS DISEASES, 73 WELBECK STREET, LONDON, W.**—There are at present 102 beds, twenty beds having been added since the beginning of this year; fifty since the beginning of the war. Nerve cases seem greatly on the increase, and certainly for many years to come treatment will be required for pensioners suffering from nerve injuries. In 1914 a scheme for re-building was being launched, and this is again under consideration, but the cost of building is about double what it was, and large donations will be urgently needed. Mr. D. D. Kirkaldy, B.A., is the Secretary.

